# Early Detection of Alzheimer’s Disease-Related Pathology Using a Multi-Disease Diagnostic Platform Employing Autoantibodies as Blood-Based Biomarkers

**DOI:** 10.3233/JAD-221091

**Published:** 2023-04-04

**Authors:** Cassandra A. DeMarshall, Jeffrey Viviano, Sheina Emrani, Umashanger Thayasivam, George A. Godsey, Abhirup Sarkar, Benjamin Belinka, David J. Libon, Robert G. Nagele, on behalf of the Parkinson’s Study Group and the Alzheimer’s Disease Neuroimaging Initiative

**Affiliations:** a Durin Technologies, Inc., Mullica Hill, NJ, USA; b New Jersey Institute for Successful Aging, Rowan University, Stratford, NJ, Department of Psychology, Rowan University, Glassboro, NJ, USA; c Department of Psychiatry and Human Behavior, Brown University, Providence, RI, USA; d Department of Mathematics, Rowan University, Glassboro, NJ, USA; e New Jersey Institute for Successful Aging, Rowan University, Stratford, NJ, Department of Gerontology & Geriatrics, Rowan University, Stratford, NJ, USA

**Keywords:** Alzheimer’s disease, antibody, autoantibodies, biomarkers, blood-based biomarkers, diagnostics, early diagnosis, mild cognitive impairment

## Abstract

**Background:**

Evidence for the universal presence of IgG autoantibodies in blood and their potential utility for the diagnosis of Alzheimer’s disease (AD) and other neurodegenerative diseases has been extensively demonstrated by our laboratory. The fact that AD-related neuropathological changes in the brain can begin more than a decade before tell-tale symptoms emerge has made it difficult to develop diagnostic tests useful for detecting the earliest stages of AD pathogenesis.

**Objective:**

To determine the utility of a panel of autoantibodies for detecting the presence of AD-related pathology along the early AD continuum, including at pre-symptomatic [an average of 4 years before the transition to mild cognitive impairment (MCI)/AD)], prodromal AD (MCI), and mild-moderate AD stages.

**Methods:**

A total of 328 serum samples from multiple cohorts, including ADNI subjects with confirmed pre-symptomatic, prodromal, and mild-moderate AD, were screened using Luminex xMAP^®^ technology to predict the probability of the presence of AD-related pathology. A panel of eight autoantibodies with age as a covariate was evaluated using randomForest and receiver operating characteristic (ROC) curves.

**Results:**

Autoantibody biomarkers alone predicted the probability of the presence of AD-related pathology with 81.0% accuracy and an area under the curve (AUC) of 0.84 (95% CI = 0.78–0.91). Inclusion of age as a parameter to the model improved the AUC (0.96; 95% CI = 0.93–0.99) and overall accuracy (93.0%).

**Conclusion:**

Blood-based autoantibodies can be used as an accurate, non-invasive, inexpensive, and widely accessible diagnostic screener for detecting AD-related pathology at pre-symptomatic and prodromal AD stages that could aid clinicians in diagnosing AD.

## INTRODUCTION

Alzheimer’s disease (AD) is a devastating, neurodegenerative disease affecting roughly 6 million people in the US [1–4]. AD-related neuropathological changes are known to begin a decade or more before emergence of hallmark symptoms [1, 4–10], making early diagnosis a challenge. This implies that, by the time tell-tale symptoms emerge and prompt neuropsychological assessments and brain imaging that can aid in diagnosing AD, a considerable amount of brain devastation may already have occurred, making it difficult to slow, stop, or potentially reverse the disease with available therapeutics. Current treatments at best only temporarily alleviate some symptoms, but do not modify pathology or disease progression, although the main target thus far has been to block amyloid-β (Aβ) deposition and thus amyloid plaque formation in the brain [11, 12]. It is critical that disease-modifying AD therapeutics, as they emerge from the pharma pipeline, can be administered as early as possible along the AD continuum, preferably at some point during the long pre-symptomatic phase, to curtail the progression of neurodegeneration and favor a successful outcome. Although many potential diagnostic tests for AD are under development, only one test requiring a cerebrospinal fluid sample obtained via spinal puncture has been approved by the FDA, and no FDA-approved blood or laboratory tests for AD yet exist that can provide a diagnosis during pre-symptomatic and prodromal (mild cognitive impairment, MCI) stages of AD. The development of accurate, noninvasive, blood-based diagnostic tests for early AD detection and monitoring for use in primary care or other frontline settings is essential to implement early treatment. Such an advancement would enable tracking of AD neuropathological and cognitive progression, make possible earlier participation in clinical trials, and inform interventions to combat this highly prevalent disease of the elderly.

The last decade has seen a surge in research aimed at developing a definitive blood test for early detection of AD. Traditional methods to diagnose AD most often involve a clinical judgement made by weighing data derived from some combination of patient history, a wide variety of simple or more extensive neuropsychological screeners and tests, diagnostic imaging, and cerebrospinal fluid (CSF) analyses of various biomarkers, such as Aβ_42_ and Aβ_40_, total tau, and various forms of phosphorylated tau (pTau) [13–22]. While some of these methods are considered “gold standards” for AD diagnosis, particularly low CSF Aβ_42_ levels for patients at MCI and amyloid PET imaging for patients at later stages of MCI and mild AD dementia, they are expensive, invasive, require highly skilled personnel to perform and evaluate these tests, and are largely inaccessible to most people throughout the world. Recently, the FDA approved the first *in vitro* diagnostic test for early detection of amyloid plaques in CSF associated with AD, intended for use in patients aged 55 years and older with cognitive impairment who are being evaluated for AD and other potential causes of cognitive decline [23–25].

Physicians are well-aware of the need for a simple, non-invasive, and inexpensive blood test to diagnose AD. Recent advancements in blood-based AD diagnostics have brought exciting potential tests to the field that involve measurements of the Aβ_42_/Aβ_40_ concentration ratio, a conformational variant of U-p53 and detection of phosphorylated versions of tau proteins, such as pTau181 and pTau217, and neurofilament light (NfL) [22, 26–34]. While these tests represent important advancements and provide a promising direction for the field of AD diagnostics, some bypass the long pre-symptomatic phase and are limited to later symptomatic stages (prodromal and more advanced stages along the AD continuum). Thus, there remains a need for a simple, non-invasive, and inexpensive blood test to diagnose AD at the earlier stages through detection of early AD-related neuropathological processes.

Nearly a decade ago, in a study of sera of 166 individuals using human protein microarrays, we showed that nearly all possessed many thousands of IgG autoantibodies (aABs) in their blood, prompting the suggestion that the function of this newly discovered aAB system is to clear debris from the blood and lymph on a day-to-day basis [35, 36]. Evidence in support of this function comes from two observations. First, in overall healthy people, individual aAB profiles can be remarkably stable, sometimes over a period of many years [37]. Second, certain aABs are selectively increased in the blood in response to the presence of disease and, importantly, these increases were consistently observed in people with the same disease. These findings led us to propose that the presence of disease triggers consistent, disease-associated changes in aAB profiles that reflect disease-associated changes in the debris profile exhibited in the blood as a result of ongoing pathological changes. Further, we speculated that detection of disease-associated increases in levels of autoantibodies in blood could be used to diagnose multiple diseases at early-stages, perhaps even before people are aware of their disease. To test this possibility, we initially used human protein microarrays to demonstrate that increased expression of certain aABs in the blood and CSF has diagnostic utility as highly accurate, sensitive, and specific biomarkers of the pathological processes associated with neurodegenerative diseases, including prodromal AD (MCI due to AD) with low CSF Aβ_42_ levels, mild-moderate AD dementia, both early-stage and mild-moderate Parkinson’s disease (PD), and multiple sclerosis [35, 36, 38–42].

More recently, additional research and development has led to the migration of our assay to a more feasible, high throughput, Luminex magnetic bead-based platform. In the present study, we sought to establish proof-of-principle for a new multiplex blood test involving the use of a small panel of aABs as blood-based biomarkers for detection of early AD-related neuropathological processes. This test includes a previously identified panel of eight aAB biomarkers, five derived from studies on prodromal AD (MCI) participants in the Alzheimer’s Disease Neuroimaging Initiative (ADNI) with confirmed low CSF Aβ_42_ levels, indicating a high likelihood of ongoing brain amyloidosis and eventual progression to AD dementia, and three derived from mild-moderate AD participants from ADNI. Our objective was to determine the overall accuracy and utility of this test for the blood-based detection of AD-related neuropathological processes in individuals at pre-symptomatic, prodromal, and more advanced stages of AD. Results demonstrate that increased levels of these eight disease-associated autoantibodies in the blood are useful as diagnostic biomarkers of the presence of AD-related pathology, distinguishing not only subjects with prodromal or more advanced stages of AD from non-AD controls, but also individuals at the pre-symptomatic stage of AD (i.e., cognitively normal individuals without subjective cognitive or memory decline who transitioned several years later to confirmed prodromal and later AD stages) with high overall accuracy, sensitivity, and specificity.

## METHODS

### Study population

We obtained banked serum samples from independent cohorts collected from participants enrolled in clinical studies [ADNI, New Jersey Institute for Success Aging’s (NJISA) Memory and Aging Program (MAP), and the Parkinson’s Study Group] and from commercial sources. Serum from 64 confirmed pre-symptomatic AD participants, 71 with MCI due to AD with confirmed low CSF Aβ_42_ levels, and 24 with mild or moderate AD dementia were obtained from ADNI. Twenty-six additional MCI and 7 AD patient samples were obtained from the NJISA MAP Program (Stratford, NJ). Sera from 106 healthy, non-demented control subjects were obtained from Reprocell USA Inc. (Beltsville, MD). Twelve early-stage PD samples were obtained from the Parkinson’s Study Group (Boston, MA). Eighteen stage 0–2 breast cancer serum samples were obtained from Asterand Bioscience, Inc. (Detroit, MI). Cohort descriptions can be found in the Supplementary Methods. All blood samples were handled using standard procedures. Demographic characteristics of the study population are listed in Table 1. The use of serum samples in this study was approved by the Rowan University Institutional Review Board (Pro2016001175 and Pro2012002275).

**Table 1:** Subject demographics

Case Demographics (*n* = 192)					
	ADNI	ADNI Prodromal	ADNI Mild-	Other Cohort	All Cases
	Preclinical AD	AD (MCI)	moderate AD	MCI/AD	(*n* = 192)
	(*n* = 64)	(*n* = 71)	(*n* = 33)	(*n* = 24)
Age Avg. (Std. Dev.)	76 (±6)	73 (±8)	74 (±7)	75 (±9)	75 (±7)
Sex (Male %)	59.4%	54.9%	30.3%	58.3%	52.6%
Ethnicity
-Asian (%)	1.6%	4.2%	0.0%	NA	2.5%
-Black (%)	7.8%	1.4%	0.0%	NA	3.8%
-Hispanic (%)	1.6%	2.8%	0.0%	NA	1.9%
-White (%)	89.1%	91.5%	100.0%	100.0%	91.8%
ApoE Proteotype
-E2/E3 (%)	6.3%	1.4%	0.0%	NA	3.1%
-E2/E4 (%)	3.1%	0.0%	0.0%	NA	1.3%
-E3/E3 (%)	54.7%	29.6%	29.2%	NA	39.6%
-E3/E4 (%)	29.7%	53.5%	41.7%	NA	42.1%
-E4/E4 (%)	6.3%	15.5%	29.2%	NA	13.8%
MMSE Avg. (Std. Dev.)	29 (±1)	27 (±2)	24 (±2)	NA	27 (±2)
CSF Aβ42 Avg. (Std. Dev.)	182 (±56)	135 (±32)	141 (±45)	NA	152 (±48)
CSF Tau Avg. (Std. Dev.)	78 (±35)	119 (±53)	108 (±42)	NA	104 (±49)
CSF pTau Avg. (Std. Dev.)	31 (±17)	44 (±15)	38 (±12)	NA	39 (±17)
Control Demographics (*n* = 136)					
	Cognitively	Non-	Neurodegenerative	All Controls	
	Normal	neurodegenerative	Control –PD	(*n* = 136)	
	Control	Control - Breast	(*n* = 18)
	(*n* = 106)	Cancer	(*n* = 12)
Age Avg. (Std. Dev.)	56 (±12)	47 (±6)	60 (±9)	55 (±11)	
Sex (Male %)	50.9%	0.0%	33.0%	42.6%	
Ethnicity (White)	100.0%	100.0%	100.0%	100.0%	

The number of individuals (n), age, gender, and race are listed for each case and control group. For ADNI samples, ApoE proteotype, MMSE, and CSF Aβ_42_, tau, and pTau are included as additional data.

### Pre-analytical serum processing

Blood collection and serum pre-processing was similar among all cohorts. ADNI, Durin Technologies Inc., Reprocell, and Parkinson’s Study Group blood samples were collected in red top tubes (BD 367820), allowed to sit at room temperature for at least 15 min to clot, centrifuged, aliquoted, and frozen at –80°C. Asterand Bioscience Inc. samples were collected in red tiger top serum separator tubes (BD 367985), allowed to sit at room temperature for at least 30 minutes to clot, centrifuged, aliquoted, and frozen at –20°C or cooler. Additional processing information for each sample cohort can be found in the Supplementary Methods section.

### Antigens

The following recombinant human antigens were coupled to Luminex xMAP^®^ Microspheres: a custom made IGL-MGC31944 (Custom R&D/Biotechne), HSH2D (Custom R&D/Biotechne), GCDH (MyBioSource - Catalog #MBS8249095), CCL19 (MyBioSource - Catalog #MBS203647), LGALS1 (Galectin-1) (Novus - Catalog #NBP2-76255), DNAJC8 (Novus - Catalog #H00022826-P01), ICAM-4 (Abnova - Catalog #H00003386-G01), and a recombinant Rabbit Anti-Human Kappa Light Chain antibody (Abcam - Catalog #ab195576) (Table 2). Proteins with buffers incompatible with the coupling chemistry were washed in 1xPBS and concentrated using protein concentrators (Pierce - Catalog #88516) before coupling.

**Table 2:** Panel of eight AD-related aAB biomarkers

Database ID	Protein Name
BC022098.1	cDNA clone MGC:31944 IMAGE:4878869 (IGL-MGC31944)
NM_032855.1	hematopoietic SH2 domain containing (HSH2D)
NM_006274.3	chemokine (C-C motif) ligand 19 (CCL19)
NM_000159.4	Glutaryl-Coenzyme A dehydrogenase, nuclear gene encoding mitochondrial protein, transcript variant 1 (GCDH)
NM_002305.4	Lectin, galactoside-binding, soluble, 1 (galectin 1) (LGALS1)
NM_014280.3	DnaJ homolog subfamily C member 8 (DNAJC8)
NM_001544.5	Intercellular adhesion molecule 4 (Landsteiner-Wiener blood group) transcript variant 1 (ICAM4)
n/a	Anti-Human Kappa Light Chain Antibody

Database identifiers and descriptions of the eight AD-related aAB biomarkers.

### Microsphere-antigen coupling

Microsphere-antigen coupling was carried out using the Luminex xMAP^®^ Antibody Coupling (AbC) Kit (40-50016) according to manufacturer’s recommendations. All antigenic proteins were coupled at 25pmol/million beads. Coupled beads corresponded to Luminex xMAP^®^ bead regions 12 (IGL-MGC31944/BC022098.1), 18 (HSH2D), 29 (Anti-Kappa), 33 (GCDH), 36 (CCL19), 44 (LGALS1), 46 (DNAJC8), and 48 (ICAM4). Antigen coupling was confirmed by testing serial dilutions of an in-house control human serum standard and/or antigen-specific antibodies.

### Assay procedure

2,500 beads/region were combined with 50μl bead mix in each well of a Costar 96 Well Plate (Catalog #3912). 50μl of participant serum, diluted 1/50 in phosphate-buffered saline (PBS TBN), was added to each well and mixed for 30 min at 37°C with shaking on an Eppendorf Thermomixer FP at 650 rpm. Samples were washed 3x with 80μl PBS-TBN using a BioTek 405 TS plate washer. 100μl of Phycoerythrin (PE) antibody (0.5 mg/ml) was added to each well and incubated for 20 min at 37°C with shaking. Samples were again washed 3x with 80μl of PBS-TBN, resuspended in 100μl PBS-TBN, and analyzed using a Luminex FlexMap3D instrument with count volume set to 50μl and the minimum bead count set at 50. All samples were run in duplicate and averaged to obtain final working values. Samples with a Coefficient of Variation (CV%) greater than 15% were discarded. Final inter- and intra-assay CV% were calculated at 10.4% and 4.9%, respectively.

### Statistical and graphical analysis

AD and healthy non-cognitively impaired control subjects were randomly split into Training and Testing Sets such that both sets contained participants of roughly equal age and sex distribution. All PD and breast cancer subjects were relegated to the Training Set. The Training Set consisted of 34 pre-symptomatic AD, 37 MCI, and 13 mild and moderate AD from ADNI, 12 MCI and 6 AD from the NJISA MAP cohort, 52 non-demented controls, as well as 12 PD and 18 breast cancer samples to represent neurodegenerative and non-neurodegenerative disease controls, respectively. The remaining samples were relegated to the Testing Set and included 30 pre-symptomatic AD, 34 MCI, 11 mild and moderate AD from ADNI, 14 MCI and 1 AD from the NJISA MAP cohort, and 54 non-demented controls. Sample grouping between the Training and Testing Sets can be found in Supplementary Figure 1.

The predictive probability model using eight biomarkers (cDNA clone MGC:31944 IMAGE: 4878869, HSH2D, GCDH, CCL19, LGALS1, ICAM4, DNAJC8, anti-IgG Kappa light chain antibody) and age as a covariate for all stages of AD represented was developed and optimized using only subjects from the Training Set and randomForest; no testing datasets were used to tune hyperparameters or optimize the final RF predictive model in any way (*RF*; v 4.6–10) in *R* (v 4.0.0) (The R Foundation for Statistical Computing, https://www.rproject.org/) [43]. The final model derived from the Training Set subjects was used to predict the probability of AD-related pathology in the Testing Set subjects. This probability was reported as the Alzheimer’s disease probability score (ADPS). An overview of the process can be found in Supplementary Figure 2. Receiver operating characteristic (ROC) curves were calculated using *R* packages ROCR (v 1.0–11) and pROC (v 1.1.18) [44], and the probability of being disease-positive is reported as a function of ROC sensitivity and specificity for each model. Additional R packages used in data analysis and visualization included ggplot 2 (v.3.3.6), and epiR (v 2.0.52).

### Calculation of the Alzheimer’s disease probability score

Samples in each of the Testing Sets were classified as either AD or a control using a percent probability output ranging from 0–100, known as the Alzheimer’s disease probability score (ADPS). The ADPS represents the fraction of trees in the forest that vote for a certain class (i.e., AD or control). Using the ADPS, classification as either AD or control was based on a specific cutoff threshold derived using ROC curves to determine the optimal cutoff value corresponding to the largest Youden’s J Statistic (sensitivity + specificity –1). All samples with a probability score above the threshold were classified as AD, and all samples falling below the threshold were classified as controls.

## RESULTS

### Serum IgG aAB biomarkers can detect AD-related pathology in patients with pre-symptomatic, prodromal, and more advanced AD

Our previous studies using human protein microarrays described a small group of aAB biomarkers that could be used in an assay to identify patients with prodromal AD (MCI), confirmed with low CSF Aβ_42_ levels, with high overall accuracy [40]. The latter is consistent with the presence of brain amyloidosis and a high likelihood of later progression to AD [17, 45–47]. Here, we migrated this assay to the Luminex magnetic bead platform, and utilized a panel of eight previously identified blood-borne IgG aAB biomarkers comprising four prodromal AD (MCI) biomarkers (cDNA clone MGC:31944 IMAGE: 4878869, HSH2D, GCDH, CCL19), three mild-moderate AD biomarkers (LGALS1, ICAM4, DNAJC8) from our earlier studies (Table 2), as well as an anti-IgG Kappa light chain antibody to measure individual IgG levels [38, 40]. Our goal was to determine if we could distinguish patients at multiple points along the early AD continuum from non-demented controls in a single test. This study had 328 participants, including 64 cognitively normal participants who later progressed to MCI/AD (here referred to as pre-symptomatic AD), 71 with prodromal AD (MCI), and 24 with mild-moderate AD, all from ADNI, along with 33 MCI/AD sera obtained from another memory clinic (NJISA MAP cohort) and 106 non-demented controls. Relative levels of the aAB biomarkers in sera were measured using a customized Luminex xMAP^®^ magnetic bead assay. Samples were separated into Training and Testing Sets, each containing roughly equal numbers of samples from patients spanning multiple stages of AD as well as non-demented controls, and were evaluated for the presence of AD-related pathology using randomForest (*RF*). Additionally, the Training Set contained 12 early-stage PD samples as neurodegenerative controls, and 18 breast cancer samples as non-neurodegenerative controls in the total control group to aid in the development of the diagnostic model for detection of early AD-related pathological processes.

**Fig. 1:**
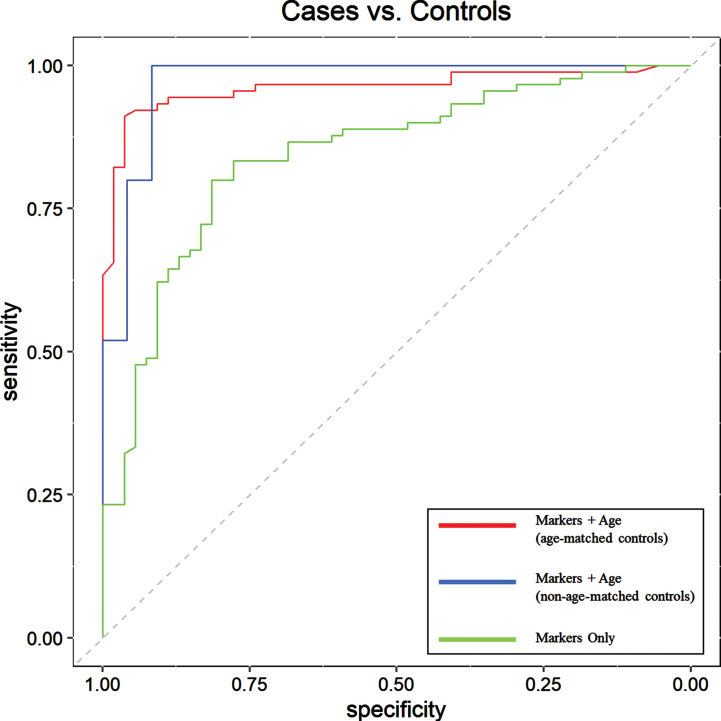
Receiver Operating Characteristic (ROC) curve assessment of aAB biomarkers for detection of AD-related pathology in Testing Set subjects; cases (pre-symptomatic, prodromal, and mild-moderate AD) (*n* = 90) versus cognitively normal controls (*n* = 54) when used alone (green line), with age as an additional parameter (blue line) in a group with non-age-matched controls, and with age as an additional parameter with a more closely age-matched control group (red line). Results show that inclusion of age as an additional parameter significantly increases overall diagnostic accuracy and, thus, the overall utility of the test. The dashed line represents the line of no discrimination. The ROC area under the curve (AUC), sensitivity, specificity, PPV, NPV, and overall accuracy values are shown in Table 3.

Using *RF* to evaluate Training Set samples (*n* = 184; 102 cases, 82 controls), a diagnostic model was created utilizing the eight selected biomarkers alone, with an out-of-bag (OOB) error of 22.3%. This model was then applied to Testing Set subjects to determine the overall classification accuracy. Subjects in the Testing Set (*n* = 144; 90 cases, 54 controls), which included pre-symptomatic, prodromal, and mild-moderate AD subjects as cases as well as healthy, non-demented controls, were classified as either positive for AD-related neuropathological processes or negative (controls), with an overall classification accuracy of 81.0%, sensitivity of 80.0%, specificity of 81.0%, positive predictive value (PPV) of 88.0%, and a negative predictive value (NPV) of 71.0%, indicating that aAB biomarker levels are concordant with the presence of ongoing AD-related pathology as was confirmed in the ADNI cohort. The diagnostic utility of this panel of eight AD biomarkers alone was also evaluated using ROC curve analysis of Testing Set subjects (Fig. 1). The ROC area under the curve (AUC) for this comparison was 0.84 (95% CI = 0.78–0.91). Diagnostic sensitivity, specificity, PPV, and NPV for the AD biomarkers when used alone to evaluate Testing Set subjects can be found in Table 3.

**Table 3:** Diagnostic utility (Testing Set subjects only) of the 8 autoantibody biomarkers alone, and with age as a covariate for predicting the probability of the presence of AD-related pathology in cases compared to controls

Testing Set Subjects	
	*n*	Threshold	AUC	Sensitivity	Specificity	PPV %	NPV %	Accuracy %
			(95% CI)	(95% CI)	(95% CI)	(95% CI)	(95% CI)
Markers + Age	49	0.65	0.97	1	0.92	0.93	1	96.0
(age-matched controls)			(0.93,1)	(0.87,1)	(0.74,0.98)	(0.77,0.98)	(0.85,1.00)
Markers + Age	144	0.56	0.96	0.92	0.94	0.97	0.88	93.0
(non-age-matched controls)			(0.93,0.99)	(0.85,0.96)	(0.85,0.98)	(0.90,0.99)	(0.77,0.94)
Markers	144	0.48	0.84	0.80	0.81	0.88	0.71	81.0
			(0.78,0.91)	(0.71,0.87)	(0.69,0.90)	(0.79,0.93)	(0.59,0.81)

Area under the curve (AUC) values at 95% confidence were generated using ROC curve analysis. Threshold values were derived using ROC curves to find the optimal cutoff value corresponding to the largest Youden’s J Statistic. Overall accuracy, sensitivity, specificity, PPV, and NPV are derived from probability data with 95% confidence intervals generated using the Wilson score method for binominal proportions.

### Inclusion of age as a covariate improves model performance and detection of AD-related pathological processes

**Fig. 2:**
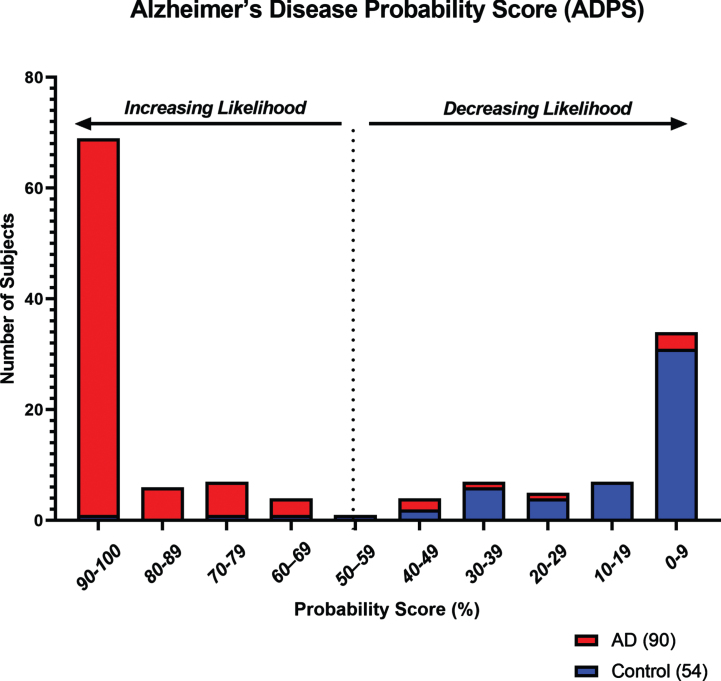
Histogram showing the distribution of Alzheimer’s Disease Probability Scores (ADPS) in Testing Set subjects (*n* = 144) for increasing or decreasing likelihood of the presence of AD-related pathology. Based on a scale of 0–100, a score of 56 or greater indicates a higher likelihood of the presence of AD-related pathology, while a score of 55 or lower indicates a reduced likelihood.

Age has been a long-established risk factor for AD [48]. Here, we examined whether adding subject age as a covariate in *RF* analysis significantly improved model performance and overall diagnostic accuracy. Addition of age as a continuous variable was found to improve the diagnostic model, resulting in a decrease of the OOB error from 22.3% to 8.2%. Overall accuracy in the Testing Set subjects was improved from 81.0% to 93.0%, and the ROC AUC from 0.84 to 0.96 (95% CI = 0.93–0.99), and had a sensitivity of 92.0%, specificity of 94.0%, PPV of 97.0%, and NPV of 88.0% (Table 3). ROC AUC comparisons with the addition of age as a covariate are shown in Fig. 1. Furthermore, using *RF* analysis, an ADPS ranging from 0–100 was calculated for predicting the likelihood of the presence of ongoing AD-related pathology as indicated by our panel of eight biomarkers and accompanying age covariate data. Based on this model, a score of 56 or greater indicates a higher likelihood for the presence of AD-related pathological processes, while a score of 55 or lower indicates a reduced likelihood. The probability score distribution for Testing Set subjects is shown in Fig. 2.

### Performance of the aAB biomarker panel in an age-matched cohort

Due to the progressively increasing prevalence of AD in aging adults, as well as the fact that neurodegenerative changes associated with this disease can begin up to two decades before the onset of clinical symptoms, the task of identifying healthy and appropriately age-matched control subjects lacking early stages of AD pathology can be fraught with potential error [49, 50]. This is particularly problematic for tests that are highly sensitive. In our Testing Set described above, we purposely used a control population that was roughly twenty years younger than our AD sample population to minimize the likelihood of the presence of early AD-related pathological changes in the controls. To demonstrate that our chosen aAB panel is not simply classifying patient samples largely based on age, we tested a closer age-matched control population by creating an additional Testing Set utilizing control samples from our original Testing Set that were obtained from individuals aged 60 years and older. Subjects in this new age-matched Testing Set (*n* = 49; 25 cases, 24 controls) included pre-symptomatic, prodromal, and mild-moderate AD samples with an average age of 71 as well as healthy, non-demented controls with an average age of 66. These samples were classified as either positive for AD-related pathology or controls using our panel of eight aAB biomarkers and age as a covariate, with an overall classification accuracy of 96.0%, sensitivity of 100.0%, specificity of 92.0%, PPV of 93.0%, and NPV of 100.0% (Table 3). This demonstrates the high sensitivity and specificity of our biomarker panel when used with closely age-matched subjects, with results comparable to the overall accuracy obtained using the non-age-matched Testing Set described above. The diagnostic utility of these biomarkers was also evaluated using ROC curve analysis (Fig. 1). The ROC area under the curve (AUC) for this comparison was 0.97 (95% CI = 0.93–1).

### aAB biomarkers can detect the presence of AD-related pathology in prodromal and later stages of AD

To further confirm the utility of our panel of eight biomarkers in accurately detecting early stages of ongoing AD-related pathological processes as well as later stages, we evaluated how many prodromal AD subjects with low CSF Aβ_42_ levels and mild-moderate AD samples in the Testing Set were correctly classified compared to controls. Using *RF* logic derived from Training Set samples based on our chosen aAB biomarkers and age covariate, 31 of 34 prodromal and all 11 mild-moderate ADNI AD samples were correctly classified. Additionally, 10 of 13 prodromal and 2 of 2 mild-moderate AD subjects from an additional cohort, the Memory Assessment Program at the New Jersey Institute for Successful Aging, were also correctly classified using the same strategy. This data suggests that our overall diagnostic strategy of including eight aAB biomarkers plus age as a covariate is robust, correctly identifying 87.2% of all prodromal AD and 100% of mild-moderate AD subjects across two independent cohorts with high overall accuracy, sensitivity, and specificity. Importantly, sera from all prodromal AD participants obtained from ADNI came from individuals with low CSF Aβ_42_ levels, consistent with the presence of ongoing early-stage brain amyloidosis, a hallmark pathological feature of early stages of AD [51].

### aAB biomarkers detect the presence of early AD-related pathological processes in subjects with confirmed pre-symptomatic AD

ADNI criteria of pre-symptomatic AD include those who initially enrolled as cognitively normal participants, but who several years later had transitioned to confirmed MCI due to AD or more advanced stages of AD dementia. ADNI criteria for normal controls include a) the absence of subjective cognitive concerns that are not due to the normal aging process, b) within normative expectation performance on cognitive screeners (MMSE and CDR) and tests (Logical Memory) (see https://adni.loni.usc.edu/methods/documents/ for cut-off scores), and c) no report of functional decline. We next asked if our diagnostic strategy, using the same panel of eight aAB biomarkers along with age as a covariate, was sensitive enough to detect the presence of ongoing AD-related pathology at an even earlier pathological stage, i.e., before the onset of observable clinical symptoms. To address this, we obtained sera from 64 ADNI participants at or near baseline who were originally diagnosed as cognitively normal based on neuropsychological assessments and normal CSF Aβ_42_ levels, but who later transitioned to either prodromal AD or a more advanced mild-moderate AD. We classified these participants as pre-symptomatic AD, and individuals in this group transitioned from cognitively normal to a diagnosis of MCI due to AD within an average of 48.3 months (median = 47.5 months) after entry into the study as cognitively normal controls. Again, using the *RF* logic derived from Training Set samples based on our eight chosen aAB biomarkers and the age covariate, 29 of 30 pre-symptomatic ADNI participants in the Testing Set were correctly identified as having AD pathology, demonstrating a 96.6% sensitivity for pre-symptomatic detection of AD-related pathological processes (Table 4).

**Table 4:** Breakdown of the probability score analysis in the Testing Set subjects using the panel of eight aAB biomarkers and age covariate in each AD-related pathological group and the non-demented control group

Testing Set Subjects	
Correctly Classified	*n*	ADNI Pre-symptomatic	ADNI MCI	ADNI MMAD	NJISA MAP MCI	NJISA MAP AD	NDC
Markers + Age (age-matched controls)	49	7/7	11/11	2/2	4/4	1/1	22/24
Markers + Age (non-age-matched controls)	144	29/30	31/34	11/11	10/13	2/2	51/54
Markers	144	23/30	29/34	11/11	8/13	2/2	45/54

## DISCUSSION

Using sera from ADNI participants and other cohorts, we examined the utility of eight selected IgG aABs; a combination of four prodromal AD (MCI) biomarkers, three mild-moderate AD biomarkers, and an anti-IgG Kappa light chain antibody, for detecting early AD-related pathology at pre-symptomatic, prodromal, and mild-moderate AD stages using a Luminex magnetic bead-based system. Most of these aAB biomarkers were selected based on their performance in previous biomarker discovery studies using human protein microarrays carried out on sera obtained from clinically well-characterized participants at prodromal and mild-moderate AD stages obtained from ADNI and Analytical Biological Systems, Inc. [38, 40]. In the ADNI cohort, the presence of early AD-related pathological processes and a diagnosis of prodromal AD (MCI) was confirmed via low CSF Aβ_42_ levels, extensive neuropsychological assessments, brain imaging, and a consensus diagnosis by ADNI investigators [40]. In the present study, additional testing of these eight aABs resulted in four main findings. First, this aAB panel identified individuals with prodromal AD and mild-moderate AD as positive for AD-related pathology and distinguished them from cognitively normal controls with high overall accuracy. Second, inclusion of age as a covariate significantly improved overall diagnostic performance at all disease stages tested. Third, the panel of aABs used also achieved detection of AD-related pathology with high overall accuracy in pre-symptomatic AD participants who originally enrolled in ADNI as cognitively normal controls, but a few years later transitioned to prodromal or more advanced AD with confirmed AD pathology.

Pre-symptomatic and prodromal AD have been particularly difficult to diagnose using current methods [9, 10, 52]. Blood-based initial screeners potentially provide an ideal and cost-effective solution for a multi-step diagnostic process that would enable a more targeted and strategic use of the more expensive and invasive CSF or PET biomarker procedures [53–56]. Some of the blood-based biomarkers under development for early diagnosis of AD include detection of Aβ_42/40_ ratios, NfL, total tau, pTau181, pTtau217, neurogranin, and aABs [29, 40, 42, 53–55, 57–59]. Many of these are showing great promise, but large-scale verification studies using standardized sample collection, storage and processing protocols, and clinically well-characterized participants are needed.

Our previous biomarker discovery studies leveraged human protein microarray technology to identify unique and consistent disease-associated changes in aAB profiles in patients with AD, PD, multiple sclerosis, psychosis, and early-stage breast cancer [35–42, 60]. For example, we found that a panel of four aAB biomarkers can readily distinguish subjects with early-stage PD from matched controls with an accuracy of 87.9% (*n* = 398 overall; sensitivity = 94.1%, specificity = 85.5%) [36]. We also studied 236 participants, including 50 with prodromal AD from ADNI confirmed via low CSF Aβ_42_ levels, neuropsychological evaluations, CSF biomarkers, and MRI and PET imaging data [46, 61], and we developed an initial panel of 10 prodromal aAB biomarkers capable of differentiating prodromal AD from non-AD controls (accuracy = 98%) with a high level of disease specificity [40].

In the present study, we tested the accuracy and utility of eight aAB biomarkers, using sera obtained from 328 individuals, for the detection of early AD-related pathological processes at pre-symptomatic, prodromal, and mild-moderate AD stages using the Luminex magnetic bead-based platform. Measurements of relative aAB levels in combination with age improved overall diagnostic accuracy in Testing Set subjects to 93.0%, and the ROC AUC to 0.96 (95% CI = 0.93–0.99). This suggests that the additional information relevant to the probability of the presence of AD-related pathology provided by inclusion of the age covariate adds to the baseline probability information provided by serum aABs alone.

A key finding reported here is that the same panel of eight aAB biomarkers, along with age as a covariate, detected the presence of early AD-related pathological changes at the pre-symptomatic AD stage. Here, we tested sera from 64 ADNI participants who were originally diagnosed as cognitively normal based on neuropsychological assessments and normal CSF Aβ_42_ levels, but later transitioned within an average of 48.3 months to either prodromal AD or a more advanced mild-moderate AD. Using the same eight aAB biomarkers, the locked *RF* logic derived from Training Set samples and the age covariate, 29 of 30 (96.6%) pre-symptomatic ADNI AD participants in the Testing Set were correctly identified. To our knowledge, this is the first blood test to accurately identify pre-symptomatic AD participants several years before the onset of clinical symptoms. Our ability to detect the presence of AD-related pathological processes pre-symptomatically in subjects initially lacking the low CSF Aβ_42_ levels, as seen in prodromal AD subjects, suggests that serum aAB biomarker levels increase before CSF Aβ_42_ levels fully drop to the low levels typical for MCI due to AD. Although it is possible that elevation of aAB biomarker levels may occur during initial phases of this downward trend in CSF Aβ_42_ levels, we cannot eliminate the possibility that aAB biomarker levels may be reflecting different aspects of ongoing AD-related pathology. The fact that the same aAB biomarkers worked well for identifying pre-symptomatic, prodromal, and mild-moderate disease stages when we combined patients at different stages of the disease into a single large group supports a scenario where it is not necessary to establish independent cutoff values for each cohort or stage of the disease. This moves us closer to the goal of a single test that can detect the presence of AD-related pathology within a relatively broad range of the early AD continuum.

This study has a number of strengths. The first is that it describes a blood-based diagnostic approach using a single panel of eight aABs as blood-based biomarkers, independent of symptoms, that can be used to detect early AD-related pathological processes at multiple recognized stages along the AD continuum in multiple cohorts with high overall accuracy. Second, it confirms results of our earlier studies using a different platform (i.e., human protein microarrays) to accurately detect prodromal and mild-moderate AD in well-characterized ADNI participants, and does so with high overall accuracy, sensitivity, and specificity [38, 40, 42]. Third, for the first time, it provides strong data supporting the utility of this approach for detecting the presence of ongoing early AD-related pathology at the pre-symptomatic stage. Fourth, this approach is a multi-disease diagnostic strategy, as shown in our previous studies describing the use of specific sets of aABs to detect and diagnose early and moderate PD, multiple sclerosis, and first-episode psychosis [36, 39, 41, 60]. Fifth, unlike many proteins and lipids, IgG aABs are particularly stable in the blood, thus ensuring that their production and detection will be largely independent of circadian as well as non-circadian day-to-day variations or a short half-life in the blood. Sixth, there were no noticeable cohort-linked differences in biomarker performance, suggesting that protocol variations in blood collection, storage and shipment did not appreciably affect measurements of IgG aAB biomarker levels in serum samples, a requisite feature for widespread use under real-world conditions. Lastly, we have shown that the use of aABs as biomarkers is not platform-specific; we were able to successfully migrate our aAB biomarker technology from human protein microarrays to a Luminex magnetic bead-based platform while retaining comparable performance. The latter is more practical, cost-effective, less technically demanding, more automatable and has greater potential for widespread use, including in rural and economically disadvantaged regions.

This study also has some weaknesses. First, it is important to note that the data presented here are limited to this group of 328 subjects from multiple cohorts, and the overall racial diversity in these cohorts was low. Second, due to the progressive nature of AD-related pathology, which can be underway a decade or more before symptoms emerge, it is difficult to find age-matched control samples that are truly cognitively normal and also free of AD-related pathology. To minimize the strong possibility that a significant fraction of age-matched controls have variable degrees of ongoing early AD-related pathology that is not yet sufficient to elicit expression of symptoms, we chose to use a control population that was roughly twenty years younger than our disease population. Although having such an age gap could potentially introduce bias, we demonstrated that using a subset of more closely age-matched Testing Set samples (only five years apart) did not significantly affect sensitivity, specificity, and overall accuracy of our diagnostic model. In a previous study on early-stage PD, we described the use of a subset of younger control subjects in which the presence and prevalence of neuropathology is considerably reduced as a compensatory mechanism for the long pre-symptomatic phase of the disease [36]. Since some members of our biomarker panel were derived from analysis of serum samples from MCI patients with low CSF Aβ_42_ levels, inclusion of younger control subjects with presumably normal CSF Aβ_42_ levels emphasizes what an aAB profile from an individual lacking AD-related pathology should look like. Third, outside of the ADNI cohort, the memory clinic and various control cohorts used here did not have measurements of CSF Aβ_42_ levels to confirm or refute the presence of early AD-related pathology involving brain amyloidosis, although this fact makes this a good “field study” for the real-world situation. Lastly, we did not test the efficacy of the AD biomarker panel for use in distinguishing patients with MCI due to AD from others with MCI due to other causes such as cerebrovascular disease, drug side-effects, depression, excessive alcohol use, poor vascular perfusion of the brain, and neurodegeneration unrelated to AD. Additional studies are currently planned to determine the utility of our biomarkers in distinguishing subjects with MCI due AD from subjects with MCI due to other causes.

In conclusion, the Luminex magnetic bead-based analytical platform described here can accurately identify the presence of early AD-related pathology in individuals with pre-symptomatic, prodromal, and mild-moderate AD based on detection of disease-associated IgG aAB biomarkers in a single blood sample. Addition of age as a covariate to our model employing aABs contributed to the excellent performance of this blood test. The development of a relatively noninvasive, accurate blood test for use in early detection of AD-related pathological processes at pre-symptomatic, prodromal, and mild-moderate stages is a significant advancement in the field given that aAB biomarkers: 1) can reliably distinguish individuals with normal versus abnormal cognitive function and predict clinical decline even in those who are asymptomatic at baseline; 2) are minimally invasive, inexpensive, and usable in frontline or community primary care settings for screening a general population; and 3) could serve as a surrogate measure for predicting outcomes in AD and AD-related dementia treatment trials. It may enable more informed determinations of which patients in the primary care settings should undergo further, more extensive neuropsychological evaluations and more invasive and costly neuroimaging (MRI and PET) and CSF diagnostic procedures. This would be of great benefit to patients and clinical practice since early treatment has the greatest potential to bend the curves on clinical outcomes. The ability to detect AD-related pathology at earlier pre-symptomatic and prodromal (MCI) stages will allow participants to be enrolled earlier in targeted clinical trials, and hopefully greatly facilitate monitoring of AD progression, including in those under treatment.

## Supplementary Material

Supplementary Material

## Data Availability

The data supporting the findings of this study are not publicly available due to privacy restrictions.
