# Supplementary material for: Early Detection of Alzheimer’s Disease-Related Pathology Using a Multi-Disease Diagnostic Platform Employing Autoantibodies as Blood-Based Biomarkers
Source: J Alzheimers Dis. 2023 Apr 4;92(3):1077–91. doi: 10.3233/JAD-221091 (PMC10116135; doi:10.3233/JAD-221091)
Supplement: Supplementary Material [file jad-92-jad221091-s001.pdf]

Supplementary Material

Early Detection of Alzheimer's Disease-Related Pathology Using a Multi-Disease Diagnostic Platform Employing Autoantibodies as Blood-Based Biomarkers

Supplementary Methods

Study population

We obtained banked serum samples from independent cohorts collected from participants enrolled in clinical studies (ADNI, NJISA's Memory Assessment Program, Parkinson Study Group, and commercial sources). The presence of AD-related pathology in samples from the ADNI was determined using one or more of the following methods: CSF biomarker analysis, amyloid PET imaging, and neuropsychological evaluation (Table 1). Because this was a retrospective sample analysis, control over preanalytical conditions during blood sample collection and processing was not possible. The processing of blood samples was similar among the different sample sources. A total of 328 participant serum samples were received and analyzed using the methods outlined below.

Clinical, biochemical, and imaging data on pre-symptomatic, MCI, and AD subjects used here were obtained from the ADNI database at https://adni.loni.usc.edu/. Our study included 64 ADNI participants who entered the study as cognitively normal participants at baseline and then later transitioned to MCI or AD over an average of 48.3 months, 71 who were diagnosed with MCI at baseline and had at least one follow-up visit, and 24 who were diagnosed with mild or moderate AD at baseline (Table 1). All ADNI participants were 55-91 years of age, non-depressed, and had a study partner able to provide collateral information regarding the participant's functioning. Individuals with a history of significant neurological or psychiatric disease, or substance misuse were excluded from the study. Per ADNI 1 criteria, normal controls included all who had Mini-Mental Status Examination (MMSE) scores between 24-30 [1], a Clinical Dementia Rating (CDR) score of 0 [2], and an absence of functional decline. MCI participants were diagnosed with 1) subjective memory complaints reported by themselves, study partner, or clinician; 2) objective memory loss defined as scoring below an education-adjusted cut-off score on delayed recall of Story A of the Wechsler Memory Scale-Revised (WMS-R) Logical Memory Test (score=8 for those with =16 years of education; score=4 for those with 8 to 15 years of education; score=2 for those with 0 to 7 years of education) [3]; 3) global CDR score

of 0.5; and 4) general cognitive and functional performance sufficiently preserved such that a diagnosis of dementia could not be made by the site physician at the time of screening. Finally, mild AD was diagnosed with a MMSE between 20 to 26, CDR of 0.5 or 1.0, and meeting NINCDS/ADRDA criteria for probable AD.

Since the inception of ADNI, there have been modifications to the diagnostic criteria. Per ADNI 2 criteria, normal controls maintained the same diagnostic criteria as in ADNI 1, with the addition of performing within normative expectations on Logical Memory II subscale (Story A Delayed Paragraph) from the WMS-R (≥9 for 16 or more years of education, ≥5 for 8 to 15 years of education, and ≥3 for 0 to 7 years of education). MCI and AD diagnostic criteria remained similar to ADNI 1 criteria. The protocols for full criteria of each ADNI phase are described elsewhere (https://adni.loni.usc.edu/methods/documents/).

The ADNI was launched in 2003 as a $60 million, 5-year, public-private partnership by the National Institute on Aging (NIA), the National Institute of Biomedical Imaging and Bioengineering (NIBIB), the Food and Drug Administration (FDA), private pharmaceutical companies, and non-profit organizations. The primary goal of ADNI has been to test whether serial MRI, PET, and other biological markers as well as clinical and neuropsychological assessments can be combined to measure the progression of MCI and early AD. Determination of sensitive and specific markers of very early AD progression is intended to aid researchers and clinicians to develop new treatments and monitor their effectiveness, as well as to lessen the time and cost of clinical trials.

The principal investigator of this initiative is Michael W. Weiner of the San Francisco VA Medical Center and the University of California San Francisco (UCSF). ADNI is the result of the efforts of many co-investigators from a broad range of academic institutions and private corporations, and subjects have been recruited from more than 50 sites across the United States and Canada. The initial goal of ADNI was to recruit 800 adults, aged 55 to 90 years, to participate in the research, with approximately 200 cognitively normal older individuals to be followed for 3 years and 200 people with early AD to be followed for 2 years. For up-to-date information, see http://www.adni-info.org.

Thirty-three AD samples were also collected from the New Jersey Institute of Success Aging Memory and Aging Program (MAP). All patients underwent an extensive neuropsychological evaluation, were examined by a social worker and a board-certified geriatric psychiatrist, and

were studied with brain MRI scans and blood serum tests. Exclusion criteria included a history of head injury, substance abuse, and major psychiatric disorders including major depression, epilepsy, B12, folate, or thyroid deficiency. This study was approved by the Rowan University institutional review board with consent obtained consistent with the Declaration of Helsinki.

The neuropsychological protocol used to classify groups included three domains of cognition: executive functions, language, and verbal memory. All test scores were expressed as z-scores derived from previously published normative data. Executive functioning measures included the Boston Revision of the Wechsler Memory Scale Mental Control subtest [4], letter fluency test ('FAS') [5, 6], and Trail Making Test-Part B [5, 7]. Language measures included the Boston Naming Test [5, 8], 'animal' fluency test [5], and WAIS-III Similarities subtest [9]. Finally, verbal memory included the CVLT-short form total learning trials, delayed free recall, and delayed recognition discriminability index [10]. All patients had an informant and presented with subjective cognitive concerns (patient or informant). Using 3-cluster classification statistical methods, a normal group and two MCI groups (amnestic-dominant and mixed-group) were generated. These MCI subtypes were then collated into one MCI group. The normal group was generally free of cognitive impairment. Comparatively, the MCI group presented with evidence of cognitive impairment relative to age and education. Of note, both the normal and MCI groups had preserved functional abilities per responses using the Lawton and Brody Scale [11]. AD was diagnosed in the presence of objective cognitive impairment and functional decline. Dementia was diagnosed via consensus of neuropsychologist, psychiatrist, and social worker. This study was approved by the Rowan University Institutional Review Board.

Individual serum pre-processing information

ADNI

Blood was collected in a red-top tube (BD 367820) and allowed to sit for at least 30 minutes at room temperature to clot. The tube is then centrifuged at 3,000 rpm for 15 min. Serum is pipetted off the clot into a prelabeled 13 ml plastic transfer tube, and then immediately capped and frozen at -80°C.

Durin Technologies Inc.

Blood was collected in a red-top tube (BD 367820) and allowed to sit for at least 30 min to clot. The tube is then centrifuged at 3,000 rpm for 15 min. Serum is transferred by pipetting off the clot into prelabeled cryovials, and then immediately capped and frozen at -80°C.

Reprocell

Blood was collected in a red-top tube (BD 367820) and allowed to sit for at least 30 min to clot. The tube is then centrifuged at 2,000 rpm for 10 min. Serum is transferred by pipetting off the clot into prelabeled cryovials, and then immediately capped and frozen at -80°C.

Parkinson's Study Group

Blood was collected in a red-top tube and allowed to sit for at least 30 min to clot. The tube is then centrifuged. Serum is transferred by pipetting off the clot into two 7 mL plastic vials, and then immediately capped and frozen at -80°C.

Asterand Bioscience Inc.

Blood was collected in a serum separator tube (SST; red tiger top) (BD 367985) and allowed to sit for at least 30 min to clot. The tube is then centrifuged at 1,300 rpm - 2,000 rpm for 10-15 min. Serum is transferred by pipetting off the clot into 1 mL cryovials, and then immediately capped and frozen at -20°C or cooler.

REFERENCES

[1] Folstein MF, Folstein SE, McHugh PR (1975) "Mini-mental state". A practical method for grading the cognitive state of patients for the clinician. *J Psychiatr Res* **12**, 189-198.

[2] Morris JC (1997) Clinical dementia rating: a reliable and valid diagnostic and staging measure for dementia of the Alzheimer type. *Int Psychogeriatr* **9 Suppl 1**, 173-176; discussion 177-178.

[3] Elwood RW (1991) Factor structure of the wechsler memory scale-revised (WMS-R) in a clinical sample: A methodological reappraisal. *Clin Neuropsychol* **5**, 329-337.

[4] Lamar M, Zonderman AB, Resnick S (2002) Contribution of specific cognitive processes to executive functioning in an aging population. *Neuropsychology* **16**, 156-162.

[5] Heaton RK, Psychological Assessment Resources (2004) *Revised Comprehensive Norms for an Expanded Halstead-Reitan Battery: Demographically Adjusted Neuropsychological Norms for African American and Caucasian Adults, Professional Manual*, Psychological Assessment Resources.

[6] Strauss E, Spreen O (1990) A comparison of the Rey and Taylor figures. *Arch Clin Neuropsychol* **5**, 417-420.

[7] Reitan RM, Wolfson D (1997) Emotional disturbances and their interaction with neuropsychological deficits. *Neuropsychol Rev* **7**, 3-19.

[8] Kaplan E, Goodglass H, Weintraub S (1983) *The Boston Naming Test. The Assessment of Aphasia and Related Disorders (2nd ed.).* Lea & Febiger, Philadelphia.

[9] Wechsler III D (1997) *WAIS-III: Administration and Scoring Manual.*

[10] Delis DC (2000) *California verbal learning test-second edition. Adult version. Manual.* Psychological Corporation.

[11] Lawton MP, Brody EM (1969) Assessment of older people: self-maintaining and instrumental activities of daily living. *Gerontologist* **9**, 179-186.

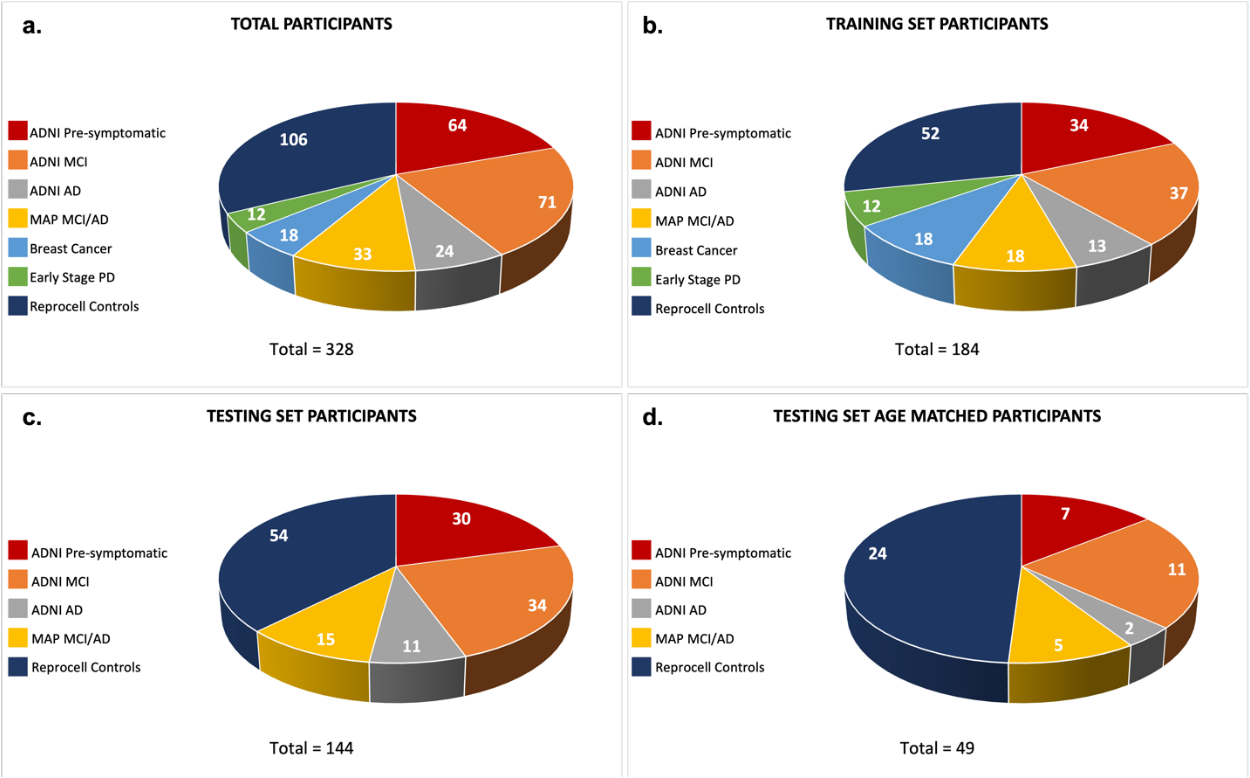

Supplementary Figure 1. Grouping of patient and control sera used in the Training and Testing Sets. a) Composition of the total participants in the study (n=328). Patients with disease were comprised of 64 pre-symptomatic MCI /AD from ADNI, 71 MCI from ADNI, 24 AD from ADNI, and 33 MCI/AD from MAP. Control patients were comprised of 18 breast cancer, 12 early-stage PD, and 106 non-demented controls from Reprocell. b) Composition of the Training Set participants in the study (n=184). Patients with disease were comprised of 34 pre-symptomatic MCI/AD from ADNI, 37 MCI from ADNI, 13 AD from ADNI, and 18 MCI/AD from MAP. Control patients were comprised of 18 breast cancer, 12 early-stage PD, and 52 non-demented controls from Reprocell. c) Composition of the Testing Set participants in the study (n=144). The patients with disease were comprised of 30 pre-symptomatic MCI from ADNI, 34 MCI from ADNI, 11 AD from ADNI, and 15 MCI/AD from MAP. The control patients included 54 non-demented controls from Reprocell. d) Composition of the Testing Set age-matched participants in the study (n=49). Patients with disease were comprised of 7 pre-symptomatic MCI from ADNI, 11 MCI from ADNI, 2 AD from ADNI, and 4 MCI and 1 AD from MAP. The control patients included 24 non-demented controls from Reprocell.

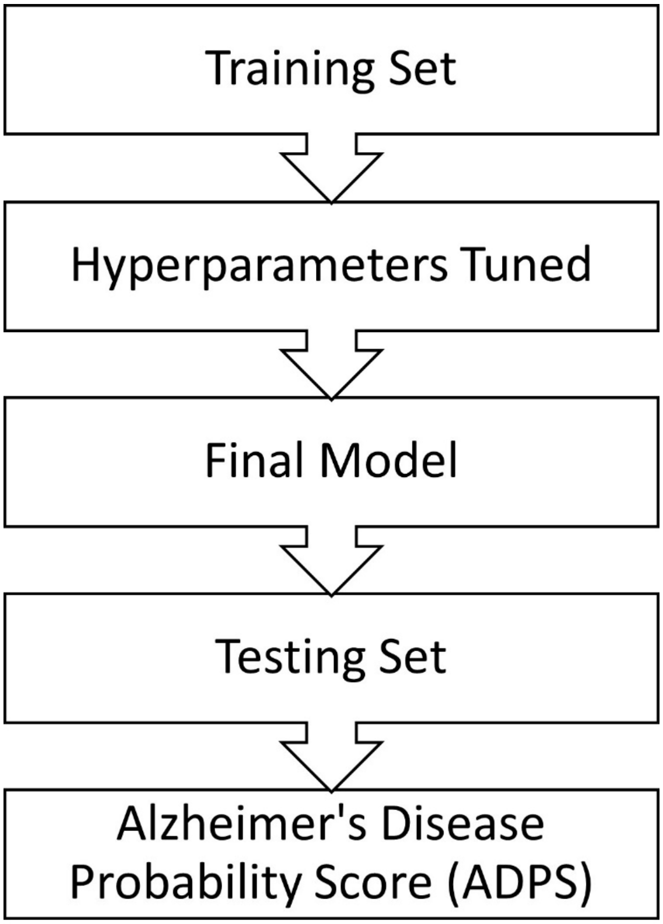

Supplementary Figure 2. Flow chart of the diagnostic model creation and testing in RF, leading to the final output as the Alzheimer's Disease Probability Score (ADPS).
